# Supplementary figures and images for: Infrared laser sampling of low volumes combined with shotgun lipidomics reveals lipid markers in palatine tonsil carcinoma
Source: Mol Oncol. 2025 Dec 23;20(6):1513–34. doi: 10.1002/1878-0261.70188 (PMC13238681; doi:10.1002/1878-0261.70188)

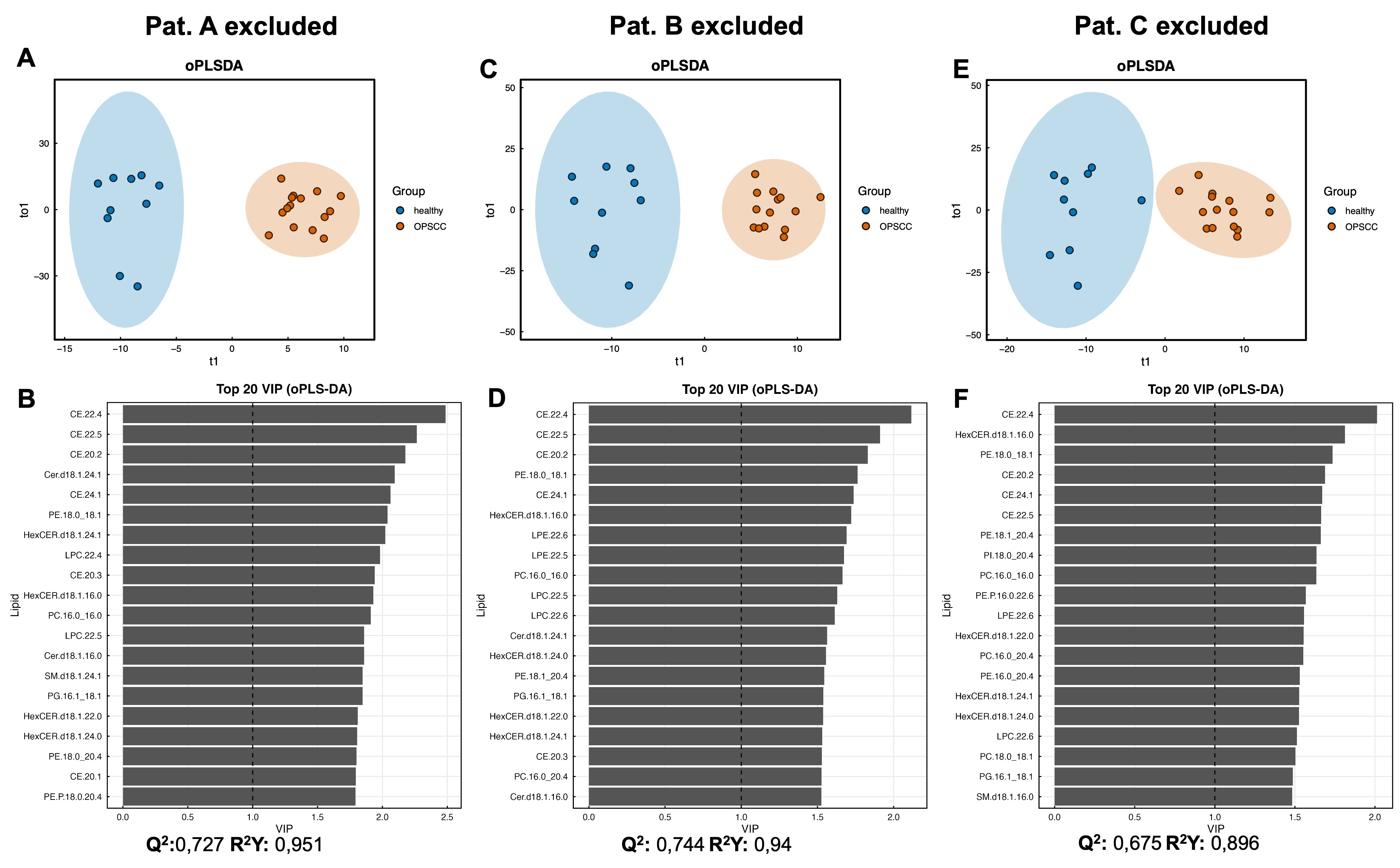

Supplement: Supplementary file 1 — Fig. S1. (A) Results of oPLS‐DA visualized as scatter plot. Data from Patient A was excluded before performing oPLS‐DA (OPSCC n = 15, healthy n = 10, 10 patients) to compare it with the oPLS‐DA model that included all patients. Technical replicates were averaged before performing oPLS‐DA. Each dot represents one sample. (B) VIP score plot of lipid species sorted after importance for tissue differentiation after exclusion of Patient A. (C) Results of oPLS‐DA visualized as scatter plot. Data from Patient B was excluded before performing oPLS‐DA (OPSCC n = 14, healthy n = 10, 10 patients) to compare it with the oPLS‐DA model that included all patients. Technical replicates were averaged before performing oPLS‐DA. Each dot represents one sample. (D) VIP score plot of lipid species sorted after importance for tissue differentiation after exclusion of Patient B. (E) Results of oPLS‐DA visualized as scatter plot. Data from Patient C was excluded before performing oPLS‐DA (OPSCC n = 15, healthy n = 10, 10 patients) to compare it with the oPLS‐DA model that included all patients. Technical replicates were averaged before performing oPLS‐DA. Each dot represents one sample. (F) VIP score plot of lipid species sorted after importance for tissue differentiation after exclusion of Patient C. [file MOL2-20-1513-s002.jpg]
